# Knowledge, confidence, and reported behaviors that promote safe water drinking among women of reproductive age

**DOI:** 10.3389/fpubh.2023.1049499

**Published:** 2023-06-26

**Authors:** Gergana Damianova Kodjebacheva, Lisa M. Lapeyrouse, Jennifer Okungbowa-Ikponmwosa, Loretta Walker, Kanday Campbell, Suzanne Cupal

**Affiliations:** ^1^Department of Public Health and Health Sciences, College of Health Sciences, University of Michigan – Flint, Flint, MI, United States; ^2^International Institute, University of Michigan – Ann Arbor, Ann Arbor, MI, United States; ^3^Institute for Social Research, University of Michigan – Ann Arbor, Ann Arbor, MI, United States; ^4^Genesee County Health Department, Flint, MI, United States

**Keywords:** women, reproductive, knowledge, confidence, behaviors, lead, water

## Abstract

**Introduction:**

Drinking lead contaminated water during pregnancy is associated with infant mortality. All women of reproductive age are advised by health agencies to adhere to healthy behaviors due to the chance of unintended pregnancy. Our objectives are to understand knowledge, confidence, and reported behaviors that promote safe water drinking and prevent lead exposure among women of reproductive age.

**Methods:**

A survey among females of reproductive age from the University of Michigan - Flint was administered. A total of 83 females who wished to become pregnant one day participated.

**Results:**

Low levels of knowledge, confidence, and reported preventative health behaviors related to safe water drinking and lead exposure prevention existed. Specifically, 71.1% of respondents (59 of 83) were not at all or were somewhat confident in their ability to choose an appropriate lead water filter. Most participants rated their knowledge on how to decrease exposure to lead during pregnancy as poor/fair. No statistically significant differences were detected between respondents residing inside and outside of the city of Flint, Michigan for most variables assessed.

**Conclusion:**

While the small sample size is a limitation, the study adds to an area of scarce research. Despite widespread media attention and resources directed toward reducing the negative health effects of lead exposure following the Flint Water Crisis, significant gaps in knowledge related to safe water drinking remain. Interventions are needed to increase knowledge, confidence, and healthy behaviors that promote safe water drinking among women of reproductive age.

## 1. Introduction

Researchers reported elevated blood lead levels among Flint, MI children on September 24th, 2015 (1). Following this discovery, residents were urged to stop drinking their tap water (1). Flint then switched from using the Flint River to the Detroit water system. Even after switching the water source, there was an additional problem of Flint’s water supply running through old, corrosive lead-lined pipes, resulting in continuous water contamination (2, 3).

Contamination of drinking water with lead is not limited to Flint, MI. Lead was discovered in drinking water in several cities across the country specifically Chicago, IL (4, 5), Milwaukee, WI (6), Baltimore, MD (7), New York City, NY (8), Newark, NJ (9), and Pittsburg, PA (10). According to data collected by the Environmental Protection Agency (EPA), 186 million people or 56% of the U.S. population consumed water with lead levels above established safety limits of 1 part per billion at some point between January 1, 2018 and December 31, 2020 (11).

Drinking lead contaminated water is especially dangerous for pregnant women, fetuses, infants, and young children (12–16). Several research studies documented an association of higher blood lead levels during pregnancy to spontaneous abortion and birth defects (12–14). Lead in pregnant women can cross the placental barrier, reducing fetal growth and increasing risk of premature birth (17). Among children, lead exposure is associated with a number of health consequences, including but not limited to impaired hearing, learning disabilities, lower IQ, and damage to the central and peripheral nervous systems (18). Studies demonstrated even low levels of exposure could cause persistent effects from childhood into adolescence and hinder educational performance in subjects such as mathematics and reading (19–23).

The negative effects of consuming lead contaminated water among pregnant women and children are clear given two U.S. water crises occurring in Washington, DC and Flint, Michigan.

During the Washington, DC Water Crisis (2000–2004), pregnant women and children were particularly susceptible to the negative health effects of lead exposure (15). Consuming lead-contaminated drinking water among women was associated with increased infant mortality and reduced birth rates (15). Similarly, in Flint, lead exposure was associated with reduced fertility rates (24) and low birthweight (25).

Given the negative consequences of drinking lead contaminated water, the EPA set the goal for lead in drinking water to zero (26), as even low levels of exposure can cause irreversible effects. Since research shows there is no safe level of exposure, healthy behaviors and proactive skills are necessary to prevent exposure to lead.

Given the dangerous effects of lead for pregnant women and their children, our study focused on investigating knowledge, confidence, and reported behaviors among women of reproductive age who would like to become pregnant one day. Since approximately half of all pregnancies in the U.S. are unintended (27, 28), the Centers for Disease Control and Prevention (CDC) recommend that all women of reproductive age adopt healthy behaviors (29). A literature review of 17 studies on the adverse influences of lead exposure on pregnancy and child health recommended that prevention of lead exposure should not only be focused on the period during pregnancy. The review emphasized that prevention of lead exposure should occur during the lifespan and especially among all women of reproductive age (30).

Preventative actions should be taken to develop the skills necessary for women of reproductive age to protect themselves and their families. As history has shown, public warnings about health effects of exposure to lead-contaminated drinking water have been reactive rather than proactive. In both the Washington and Flint Water Crises, information on how to protect oneself from lead-contaminated water surfaced after exposure occurred and elevated blood lead levels were detected (1, 15). Failure to prepare women of reproductive age in advance of such health threats can result in prolonged exposure and increased risk for negative health outcomes for women, fetuses, and families more broadly.

To minimize exposure to lead-contaminated water and promote safe water drinking practices, women of reproductive age must be aware of the need and processes for testing water at home (16, 31). Since no one can smell, taste, or see lead dissolved in water, regular testing is the only method in determining its presence (17). Selecting a correct filter to eliminate lead is also essential (16, 32). Not all filters can eliminate or decrease all contaminants. An appropriate filter for lead-contaminated water, would be one certified by the National Sanitation Foundation (NSF) to remove lead (16, 31, 32). Proper installation and regular maintenance, such as replacing filters when necessary, ensure both optimum function of filters and reduced effects from lead exposure. Additionally, women should understand the benefit of running cold water to flush their pipes and cleaning faucet aerators regularly because lead pipes, plumbing fixtures, and faucets are deemed the most common sources of contamination (26, 31). Running water for at least 1 min prior to use is an effective strategy to prevent exposure to lead (33).

The current study surveyed women of reproductive age who planned on becoming pregnant in the future. All participants resided in Genesee County, Michigan including the city of Flint residents and non-Flint residents. Flint is the largest city in Genesee County. It had lower socio-economic status compared to Genesee County as a whole (34). The City of Flint experienced the Flint Water Crisis because its water source was switched from the Detroit River to the Flint River (1). Past research found that Flint residents reported physical health deterioration (35), post-traumatic stress disorder (36), and sleep problems (37) associated with poor tap water quality during the water crisis. The source of the water in all other cities surrounding Flint in Genesee County was not the Flint River. Genesee County residents outside of the City of Flint, therefore, did not experience lead water contamination in their homes.

Given the emphasis on child health as a result of water crises as well as limited awareness of the dangers and prevention of lead water commination, one hypothesis was that women of reproductive age would report low levels of knowledge and confidence and would engage in limited reported proactive health behaviors related to safe water consumption overall in the entire sample. A second hypothesis was that educational efforts and health programming stemming from the Flint Water Crisis would result in higher levels of knowledge, skills, and reported preventive health behaviors among women of reproductive age who resided in Flint compared to those who resided outside of Flint in Genesee County.

## 2. Methods

The design was a cross-sectional one-time, online survey with close-ended questions. The Institutional Review Board of the University of Michigan approved the study. All registered students attending the University of Michigan - Flint campus were sent an email requesting participation. Only female students, 18–45 years of age who planned to become pregnant in the future were asked to respond.

The study setting was the University of Michigan-Flint located within the city of Flint, in Genesee County, Michigan (Supplementary material). Some participants were from Flint, MI and others were from outside of the City of Flint but still from Genesee County. The following are estimates from July 1, 2022 on Flint, MI and Genesee County, MI to understand the differences in socio-economic and demographic characteristics between the two areas. The percent of persons aged 25 years and older with a bachelor’s or higher degree was 22.2% in Genesee County compared to 12.1% in Flint (34). The percent of persons living in poverty was 16.3% in Genesee County compared to 35.5% in Flint. The percent of White persons was 75.0% in Genesee County compared to 34.7% in Flint. The percent of Black or African American persons was 20.3% in Genesee County compared to 56.7% in Flint in 2022 (34). The median household income in 2021 dollars was $54,052 in Genesee County compared to $32,358 in Flint (34). As the Supplementary file demonstrates, according to 2019 data, the median household income in Flint was lowest of all cities in Genesee County.

The recruitment email contained a link to the online survey. Prior to the online survey, there was a statement emphasizing that participation was voluntary and that participants could skip any items and decide to stop participation at any time. Written informed consent for participation was not required for this study because the study involved the collection of survey data and entailed no more than minimum risk to participants. Students received a total of 3 reminders to complete the survey over a 2-month period. At the end of the 2-month period, the data collection stopped.

All participants were asked to answer voluntary written socio-demographic items (Table 1). The close-ended items on knowledge, confidence, and reported behaviors were developed by a public health educator (GDK) and a county public health director (SC) based on their development of and participation in educational events for Flint residents organized by the Genesee County Health Department (Table 2). The items on knowledge, confidence, and reported behaviors were not assessed for reliability and validity. The public health educator and county public health director developed the items based on recommended strategies for prevention of lead exposure by public health agencies (16, 17, 31–33). For example, women were asked to rate their confidence in activities (i.e., choosing an appropriate lead water filter at the store, installing a lead water filter on a kitchen sink, and cleaning the aerator of their bathroom faucet) and preventive practices (i.e., running tap water for at least 1 min prior to using it) which were recommended to decrease lead exposure through contaminated water by health public health agencies (16, 17, 31–33).

**Table 1:** Survey items on socio-demographic background and self-reported knowledge, conference, and behaviors related to lead administered to participants.

Socio-demographic survey items Please enter your age in years: Please mark one or more of the following to describe yourself. *Response options: White/Caucasian; Black/African American; American Indian/Native American; Alaskan Native Asian; Native Hawaiian; Pacific Islander; other, please specify* Are you Hispanic, Latino, or Spanish origin? *Response options: Yes; No* What is your current relationship status? *Response options: single-never married; married; other committed relationship; separated; widowed; Divorced* How many children under the age of 18 do you have? *Response options: 0; 1; 2; 3; more than 3* Where do your reside? *Response options: City of Flint, Genesee County; outside of the city of Flint but still in Genesee County; other, please specify_____* Was anyone in your household/family employed at least 50 weeks out of the past 52 weeks? *Response options:* Yes; No We would like to ask the range of the total income received by all members of your household for last year. Would you say that the total combined family income before taxes was: *Response options: Less than $20,000; $20,000 to $39,999; $40,000 to $59,999; $60,000 to $79,999; $80,000 and above* Survey items on knowledge, confidence, and behaviors related to lead **Knowledge** Please rate your level of knowledge of the following issues on a scale of *poor, fair, good, very good, excellent* Knowledge about the health effects of exposure to lead in the pregnant woman during pregnancy Knowledge about the health effects of exposure to lead in children Knowledge on how to decrease exposure to lead before during and after pregnancy Knowledge about the safety of bottled water **Confidence** Please rank how confident you are on a scale of *not at all confident, somewhat confident, confident, very confident* in your ability to: Install a lead water filter on a kitchen faucet? Choose an appropriate lead water filter at the store? Clean the aerator of your bathroom faucet? **Reported behaviors** Please indicate how often you do the following with a frequency of *never, rarely, sometimes, very often, always*: Run the tap water at least 1 min? Wipe and remove your shoes before entering your place? Clear floors, windowsills, and other surfaces in your home? Wash your hands before eating?

**Table 2:** Socio-demographic characteristics of survey participants, *N* = 83.

	*N*	Percentage
**Age**		
18 to 24	58	69.9%
25 to 29	9	10.8%
30 to 34	11	13.3%
35 and older	3	3.6%
No response	2	2.4%
**Race/Ethnicity**		
White/Caucasian	62	74.7%
Black/African American	14	16.9%
American Indian/Native American	0	0.0%
Asian	4	4.8%
Native Hawaiian/Pacific Islander	0	0.0%
Other	2	2.4%
No response	1	1.2%
**Hispanic, Latino, or Spanish**		
Yes	2	2.4%
No	80	96.4%
No response	1	1.2%
**Relationship status**		
Single/never married	46	55.4%
Married/other committed relationship	34	41.0%
Separated/widowed/divorced	3	3.6%
**Children under 18**		
0	68	81.9%
1	6	7.2%
2	7	8.4%
3	1	1.2%
>3	1	1.2%
**Area of residency**		
City of Flint, Genesee county	21	25.3%
Genesee county, outside of the city of Flint	62	74.7%
Household employment (50 of last 52 weeks)		
Yes	77	92.8%
No	6	7.2%
**Annual household income**		
Less than $20,000	18	21.7%
$20,000–$39,999	19	22.9%
$40,000–$59,999	7	8.4%
$60,000–$79,999	10	12.0%
$80,000 and over	29	34.9%

Women were asked to rate their knowledge as poor, fair, very good, good, and excellent on several items related to safe water drinking. For example, they were asked to rate their knowledge on: the health effects of exposure to lead in the pregnant woman during pregnancy; how to decrease exposure to lead before, during, and after pregnancy; and any contaminants (lead and others) in tap water. Women were asked about their reported behaviors, specifically how often they ran their tap water at least 1 min before using it and how often they washed their hands before eating; response options were never, rarely, sometimes, very often, always.

Since lead exposure occurs not only through water usage but also through other means such as having dust in the home and having untidy hands while consuming food, women were asked to rate how frequently they wiped and removed shoes prior to entering their home, cleaned floors, window sills, and other surfaces in the home (38–42), and washed their hands before eating (42). Wording in items was similar to official recommendations. For example, a suggested step to prevent lead exposure according to the EPA is: “regularly clean floors, window sills, and other surfaces” (42). Therefore, the survey asked: “How often do you clean floors, windowsills, and other surfaces in your home?”

Qualtrics software was utilized to obtain the data from the online survey. Data were entered in the statistical software IBM SPSS Version 29. The percentage and number of women giving responses for the different response options were calculated for all women overall and for women residing inside and outside of Flint, MI. Response options were collapsed into fewer categories (i.e., poor/fair compared to good/very good/excellent, not at all confident/somewhat confident compared to confident/very confident, never/rarely compared to sometimes/very often/always) in the tables but examples of findings with all response categories were presented in the text of the Results as well. Two-sided Fisher’s exact test and Pearson chi-square tests were conducted to compare knowledge among women inside and outside of Flint, MI.

## 3. Results

### 3.1. Socio-demographic characteristics among participants

Among the 83 participants, 69.9% were aged 18 to 24, 74.7% were White/Caucasian, and 55.4% were single/never married (Table 2). Most (81.9%) had no children, 92.8% had someone in their household employed 50 of the last 52 weeks, and 74.4% resided outside of Flint.

### 3.2. Reported knowledge, confidence, and behaviors among participants overall

The majority of participants rated their knowledge on the following as poor/fair: the health effects of exposure to lead in the pregnant woman during pregnancy and how to decrease exposure to lead before, during, and after pregnancy (Table 3). The percentages and numbers for each response category for the item on rating one’s knowledge on the health effects of exposure to lead in the pregnant woman during pregnancy were: poor 12% (10), fair 38.6% (32), good 30.1% (25), very good 15.7% (13), and excellent 3.6% (3). Participants were slightly more confident in their ability to install a lead water filter on a kitchen sink than to choose one at the store. Specifically, the percentages and numbers for each category for the item on rating one’s confidence in the ability to choose an appropriate lead water filter at the store were not at all confident 31.3% (26), somewhat confident 39.8% (33), confident 13.3% (11), very confident 15.7% (13). The percentages and numbers for each category for the item on rating one’s confidence in the ability to install a lead water filter on a kitchen sink were not at all confident 30.1% (25), somewhat confident 34.9% (29), confident 21.7% (18), very confident 13.3% (11).

**Table 3:** Knowledge, skills, and behaviors related to lead among survey participants, *N* = 83.

Question/response	Poor/fair	Good/very good/excellent
**Please rate your level of knowledge about**	% (*N*)	% (*N*)
The health effects of exposure to lead in the pregnant woman during pregnancy	50.6% (42)	49.4% (41)
The health effects of exposure to lead in children	32.5% (27)	67.5% (56)
How to decrease exposure to lead before, during, and after pregnancy	71.1% (59)	28.9% (24)
The safety of bottled water	47.0% (39)	53.0% (44)
Question/response	Not at all confident/somewhat confident	Confident/very confident
**Please rank how confident you are in your ability to**	% (*N*)	% (*N*)
Choose an appropriate lead water filter at the store	71.1% (59)	28.9% (24)
Install a lead water filter on a kitchen faucet	65.1% (54)	34.9% (29)
Clean the aerator of your bathroom faucet	89.2% (74)	10.8% (9)
Question/response	Never/rarely	Sometimes/very often/always
**Please indicate how often you do the following**	% (*N*)	% (*N*)
Run the tap water at least 1 min before using it	63.9% (53)	36.1% (30)
Wipe and remove your shoes before entering your place	47.0% (39)	53.0% (44)
Clean floors, window sills, and other surfaces in your home	69.9% (58)	30.1% (25)
Wash your hands before eating	33.7% (28)	66.3% (55)

Over 70% rated their confidence in abilities to choose an appropriate lead water filter at the store and clean the aerator of the bathroom faucet as poor/fair. The percentages and numbers for each category for the item on rating one’s confidence in the ability to clean the aerator of the bathroom sink were not at all confident 59.0% (49), somewhat confident 30.1% (25), confident 8.4% (7), very confident 2.4% (2). The percentage of participants never/rarely running their tap water at least 1 min before using it was 63.9% (53 out of 83). Thirty-three point seven percent (28 out of 83) never/rarely washed their hands before eating.

### 3.3. Reported knowledge, confidence, and behaviors among participants residing inside and outside of Flint

No statistically significant differences in most knowledge, confidence, and reported healthy behaviors between residents inside and outside of Flint existed (Table 4). Knowledge was primarily low in both areas. For example, 81.0% (17 out of 21) of respondents in Flint rated their knowledge on how to decrease exposure to lead before, during, and after pregnancy as poor/fair compared to 87.1% (54 out of 62) outside of Flint (*p* = 0.488). There was a higher perceived skill in the ability do install a lead water filter in Flint than outside of Flint, which was statistically significant (Table 2). Specifically, 71.4% (15 out of 21) of respondents in Flint were not at all/somewhat confident in their ability to install a lead water filter on a kitchen faucet compared to 91.9% (57 out of 62) outside of Flint (*p* = 0.026).

**Table 4:** Knowledge, skills, and behaviors related to lead comparing survey participants residing inside and outside of Flint, *N* = 83.

	Inside Flint		Outside Flint		value of p*
Item/response	Poor/fair	Good/very good/excellent	Poor/fair	Good/very good/excellent	
**Please rate your level of knowledge about**	% (*N*)	% (*N*)	% (*N*)	% (*N*)	
The health effects of exposure to lead in the pregnant woman during pregnancy	47.6% (10)	52.4% (11)	51.6% (32)	48.4% (30)	0.754*
The health effects of exposure to lead in children	28.6% (6)	71.4% (15)	33.9% (21)	66.1% (41)	0.654*
How to decrease exposure to lead before, during, and after pregnancy	81.0% (17)	19.0% (4)	87.1% (54)	12.9% (8)	0.488**
The safety of bottled water	81.0% (17)	19.0% (4)	71% (44)	29.0% (18)	0.568**
Item/response	Not at all confident/somewhat confident	Confident/very confident	Not at all confident/somewhat confident	Confident/very confident	
**Please rank how confident you are in your ability to**	% (*N*)	% (*N*)	% (*N*)	% (*N*)	
Install a lead water filter on a kitchen faucet	71.4% (15)	28.6% (6)	91.9% (57)	8.1% (5)	0.026**
Choose an appropriate lead water filter at the store	71.4% (15)	28.6% (6)	88.7% (55)	11.3% (7)	0.082**
Clean the aerator of the bathroom faucet	95.2% (20)	4.8% (1)	98.4% (61)	1.6% (1)	0.444**
Item/response	Never/rarely	Sometimes/very often/always	Never/rarely	Sometimes/very often/always	
**Please indicate how often you do the following**	% (*N*)	% (*N*)	% (*N*)	% (*N*)	
Run the tap water at least 1 min before using it	61.9% (13)	38.1% (8)	64.5% (40)	35.5% (22)	0.830*
Wipe and remove your shoes before entering your place	71.4% (15)	28.6% (6)	67.7% (42)	32.3% (20)	0.099*
Clean floors, window sills, and other surfaces in your home	61.9% (13)	38.1% (8)	72.6% (45)	27.4% (17)	0.357*
Wash your hands before eating	14.3% (3)	85.7% (18)	40.3% (28)	59.7% (37)	0.029*

* Pearson chi-square test, two-sided.

** Fisher’s exact test, two sided.

## 4. Discussion

### 4.1. Summary and interpretation of findings and comparison of current results to those of prior research

One of the study hypotheses was that women of reproductive age would report low levels of knowledge and confidence and would report engaging in limited proactive health behaviors related to prevention of lead exposure overall. Indeed, we found that most women in our sample reported low levels of knowledge, confidence, and healthy behaviors. For example, 59.0% (49) of participants were not at all confident in their ability to clean a sink aerator.

The second study hypothesis was that there would be higher levels of knowledge, confidence, and reported preventive health behaviors among women of reproductive age who resided in Flint compared to those who resided elsewhere. Residents inside of Flint tended to have slightly higher knowledge, confidence, and reported preventative behaviors but these findings were not statistically significant for all variables except for installing a lead water filter on a kitchen sink. When it came to confidence in installing a lead water filter on a kitchen sink women in Flint fared better compared to those elsewhere. Specifically, among people outside of Flint, 91.9% had poor/fair confidence in installing a lead water filter on a kitchen sink compared to 71.4% of those inside Flint (*p* = 0.026). Educational efforts and provision of free lead water filters to Flint residents stemming from the water crisis may have contributed to the more positive findings in the city. Still, despite educational efforts, knowledge, confidence and behaviors were low even in Flint, MI. For example, 81.0% of participants rated their knowledge on how to decrease exposure to lead before, during, and after pregnancy as poor/fair in Flint compared to 87.1% outside of Flint (*p* = 0.488).

Overall, a greater proportion of women ranked their knowledge about the health effects of lead exposure on children better than their knowledge of the health effects of lead exposure on pregnant women, the safety of bottled water, and how to decrease exposure before, during, and after pregnancy. For example, 67.5% of women rated their knowledge on the health effects of exposure to lead in children as good/very good/excellent compared to 28.9% of women who rated their knowledge on how to decrease exposure to lead before, during, and after pregnancy as good/very good/excellent. The results on the higher knowledge on the effect of lead on children may not be surprising given the emphasis on child health and well-being during water crises. Greater efforts to educate women on the negative effects of lead on pregnancy, not only child health, are needed in Flint and beyond.

We were unable to locate a study that asked various questions related to knowledge, confidence, and behaviors with a focus on safe water drinking among either women or other populations. In a prior qualitative study, women of reproductive age recommended using and changing water filters in the home to prevent lead exposure before and during pregnancy (43). The finding on hand-washing that 33.7% (28 out of 83) never/rarely washed their hands before eating in this study is similar to that of a prior study (44). A study representative of the noninstitutionalized U.S. population aged 18 years and older found that in 2019, 44.8% of adults did not remember to wash their hands before eating at a restaurant and 37.2% did not remember to wash their hands before eating at home (44).

### 4.2. Limitations

Limitations of the study included its small sample size and the use of a non-probability sample of university students. Despite sending 3 reminders to students to complete the survey over a 2-month period, only 83 participants responded. Placing restrictions that only women who wished to become pregnant in the future limited the number of participants. The study had a small budget that did not allow for each survey participant to receive a gift card. Having a small gift card for each participant may have assisted in having a larger sample size. Most respondents reported relatively high incomes. The higher socio-economic position of respondents in our study is a limitation because the results may not be generalized to women of lower socio-economic status. Recent studies determined that low socio-economic status was a key risk factor for elevated blood lead levels in children (45). Factors associated with low socio-economic status such as low maternal knowledge of lead and poor nutrition were associated with elevated blood lead levels (46). Together, these factors increase susceptibility to lead exposure, thus contributing to higher blood lead levels in children and families of lower compared to higher socio-economic status (45).

The racial/ethnic characteristics of our sample were similar but not the same compared to those of University of Michigan – Flint students. White/Caucasian students were slightly over-represented while Hispanic/Latino students were underrepresented in our study when comparing our participants and the University of Michigan – Flint student population. Specifically, the percentage of White/Caucasian students in our sample was 74.7% compared to 67.0% for the university students (47). The percentage of Black/African American students in our sample was 16.9% compared to 12.6% for the university students. The percentage of Hispanic/Latino students in our sample was 2.4% compared to 5.4% for the university students (47).

The participants were university students who may not be representative of the population in Genesee County. Based on 2022 U.S. Census data, the percent of White persons in Genesee County was 75% (34) compared to 74.7% in our study. The percent of Black or African American persons in Genesee County was 20.3% (34) compared to 16.6% in our study. The percent of Hispanic or Latino persons was 3.9% in Genesee County (34) compared to 2.4% in our study. Black or African American persons and Hispanic or Latino persons, therefore, were underrepresented in our study when comparing to the population in Genesee County, Michigan.

Another limitation is that the study did not use an established valid and reliable survey on knowledge, confidence and reported behaviors related to lead exposure among women of reproductive age. Knowledge, confidence and behaviors were self-reported. Knowledge-based questions (such as true/false questions related to prevention strategies on lead exposure) were not asked. Yet another limitation is the study did not ask participants to differentiate if they washed their hands before cooking at home, before eating at home, before eating at a restaurant, and before coughing and sneezing. The study asked only close-ended questions and did not conduct focus groups or interviews.

### 4.3. Implications for future research

Since the current study involved only 11 close-ended survey items, future research may employ a qualitative methodology including focus groups and interviews to ask for the deeper thoughts of participants. Questions may ask not only about knowledge, confidence, and behaviors but also about suggestions on how to promote safe water drinking and prevent lead exposure. Focus groups and interviews can be conducted not only with women but also with partners and family members.

Another implication for future research is the need for interventions to advance knowledge, skills, and behaviors that promote safe water drinking and prevent lead exposure. A systematic review of interventions to reduce exposure to lead identified only 2 interventions in the U.S. (30). The literature review concluded there is an urgent need for: “research into the effectiveness of interventions to reduce lead exposure from consumer products and drinking water (30).” The studies in the literature review focused on measuring blood lead levels in children before and after the interventions (30). These prior interventions that sought to educate women on the sources of lead, negative health effects of lead, prevention of lead exposure did not assess the knowledge of participants (30). In one of the interventions, 594 mothers were randomly assigned to an intervention and control groups (22). Women in the intervention group received 20 bi-weekly educational sessions over the course of 1 year and quarterly refresher sessions for 2 years afterwards. The educational sessions incorporated information on lead sources (specifically paint, dust, water, soil, and risks from home repairs and remodeling), health influences of lead exposure, and prevention strategies such as household cleaning, hand washing, safe water use, and nutritional changes. Intervention children had an increased likelihood to have blood lead levels <10 μg/dL compared to controls (81% vs. 73%; *p* = 0.08) (22). A valuable aspect of the intervention was that the educator was of the same ethnicity as the mother who was participating in the study. Future interventions should also have ethnically-tailored interventions especially for African Americans since they are at a higher risk of lead exposure (48–51).

Since only 3.6% (3 women) rated their knowledge about the health effects of exposure to lead in the pregnant woman during pregnancy as excellent, 15.7% (13) as very good, 30.1% (25) as good, 38.6% (32) as fair, and 12.0% (10) as poor, interventions at the physician office and media campaigns to promote safe water drinking will be valuable. Education on cleaning bathroom sink aerators and choosing an appropriate filter at the store are especially needed because when comparing responses to confidence items, participants were least confident in their ability to clean the aerator of the bathroom sink followed by the ability to choose an appropriate filter at the store. A prior study involving open-ended questions and focus groups suggested the use of safe water drinking educational pamphlets in physician offices. During 3 focus groups with 27 women of reproductive age, participants suggested a national media campaign to use messages such as “Warrior Story,” “Follow these precautions to have a successful pregnancy,” and What type of water are you drinking?” to increase knowledge on safe water drinking (29). Such interventions may help create a culture of healthy behaviors for women. In turn, women can adopt this culture of behaviors regardless of the existence of public warnings regarding water contamination. Involving people of lower socio-economic status in health promotion interventions is especially needed because most people affected by water contamination in Flint were located in poor neighborhoods (52).

Interventions are also needed to stimulate and remind individuals to wash their hands to reduce contracting infectious diseases. In past research, strategies included hygiene education, provision of handwashing supplies, and media campaigns (53). Men have an increased likelihood not to wash their hands before eating (44). The current study implies that women also need hand washing interventions. Such interventions will be valuable to protect not only women but also their fetuses if pregnant.

### 4.4. Conclusion and recommendations

We found that most women in our sample reported low levels of knowledge, confidence, and healthy behaviors related to safe water drinking and lead exposure prevention. Residents inside of Flint tended to have slightly higher knowledge, confidence, and reported preventative behaviors but these findings were not statistically significant for all variables except for installing a lead water filter on a kitchen sink. Findings of the current research highlight the need for surveys and interventions with larger sample sizes to understand and improve knowledge, confidence, and behaviors related to minimizing lead exposure. Education on the negative effects of lead on pregnancy outcomes should be included in the interventions. Demonstration on how to choose an appropriate filter, install a filter, and clean the aerator of the bathroom faucet will be valuable. Since a limitation of the current investigation was that most participants were of higher socio-economic status, future studies should seek to assess and improve knowledge, confidence, and behaviors among women of lower socio-economic status. The effectiveness of interventions, not only on safe water drinking but also on blood lead levels and pregnancy outcomes, should be investigated.

## Data availability statement

The raw data supporting the conclusions of this article will be made available by the authors, without undue reservation.

## Ethics statement

The studies involving human participants were reviewed and approved by the IRB: University of Michigan Institutional Review Board. Written informed consent for participation was not required for this study in accordance with the national legislation and the institutional requirements.

## Author contributions

GK and SC conceived and designed the study and data collection tools. GK, JO-I, LW, KC, and SC collected the data. GK, LL, JO-I, LW, KC, and SC performed the analysis and reviewed the final paper. All authors contributed to the article and approved the submitted version.

## Funding

The study was funded by an internal grant by the College of Health Sciences, University of Michigan – Flint. The first author was supported by an academic fellowship.

## Conflict of interest

The authors declare that the research was conducted in the absence of any commercial or financial relationships that could be construed as a potential conflict of interest.

## Publisher’s note

All claims expressed in this article are solely those of the authors and do not necessarily represent those of their affiliated organizations, or those of the publisher, the editors and the reviewers. Any product that may be evaluated in this article, or claim that may be made by its manufacturer, is not guaranteed or endorsed by the publisher.
