# Supplementary figures and images for: Knowledge, confidence, and reported behaviors that promote safe water drinking among women of reproductive age
Source: Front Public Health. 2023 Jun 26;11:1049499. doi: 10.3389/fpubh.2023.1049499 (PMC10331607; doi:10.3389/fpubh.2023.1049499)

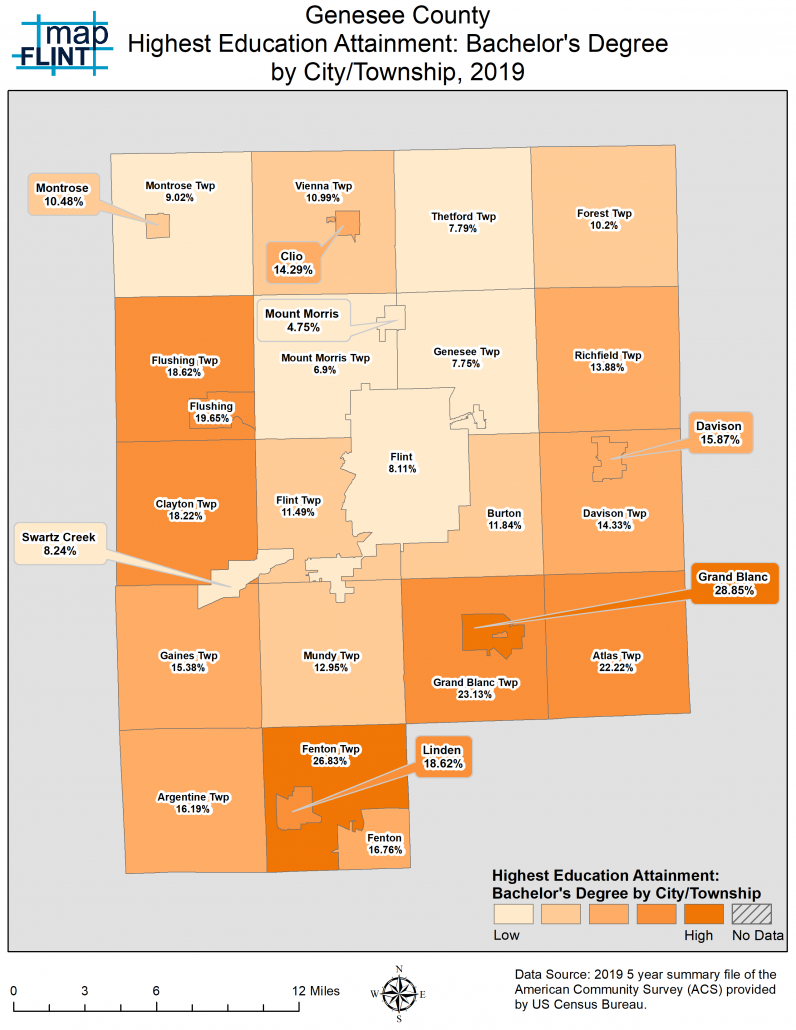

Supplement: Supplementary file 1 [file Image_1.PNG]
